# Commentary: Mental Health in Sport (MHS): Improving the Early Intervention Knowledge and Confidence of Elite Sport Staff

**DOI:** 10.3389/fpsyg.2017.01209

**Published:** 2017-07-18

**Authors:** Amelia Gulliver

**Affiliations:** The Centre for Mental Health Research, The Australian National University Canberra, ACT, Australia

**Keywords:** athletes, elite sport, early intervention, mental health, mental illness, mental health literacy, stigma

## Context

Elite athletes have comparable levels of mental health problems to the general community, yet many do not seek professional help. Previous research has focused on increasing help-seeking in athletes by targeting stigma and mental health literacy in the athletes themselves (Gulliver et al., 2012). Increased training and engagement of coaches may assist in facilitating athletes' access to professional mental health care (Gulliver et al., 2012). However, limited research has been conducted to date that aims to increase knowledge around mental health problems for coaches and support staff.

## Methods

A total of 166 elite sport staff members (aged 22–66 years, 50.0% female) including coaches, trainers, support staff, and service providers (nutritionists, physiotherapists) working in the Australian high performance network were recruited. The study used a quasi-experimental design with eight workshops divided into four experimental (85 participants) and four waitlist control (81 participants) groups. Outcome measures were the 11-item versions of the depression (D-Lit) and anxiety (A-Lit) literacy questionnaires (ranges 0–11), and a measure developed for the purposes of the study comprising four items assessing the participants' confidence in recognizing, reaching out to, referring to a professional, and finally supporting someone with a mental health problem. Data analysis methods were intention-to-treat using maximum likelihood estimation (MLE) methods.

## Findings

The depression and anxiety literacy of the coaches and support staff, as well as their confidence in helping someone with a mental health problem was significantly improved by 1–2 points at post-intervention. Confidence in assisting people with mental health problems improved on average from “moderately” to “quite a bit.” Lack of randomization was noted as a key limitation by the authors. Given the pre-existing workshop locations, cluster randomization could perhaps have been considered.

## Commentary

The current study by Sebbens et al. is the first of its kind and demonstrated small but significant increases in depression and anxiety literacy in coaches and other support staff. Sebbens et al.'s study is important and necessary research that additionally contributes vital dialog in normalizing the existence and treatment of mental health problems in elite athletes.

One of the key issues identified in Sebbens et al.'s study was the low rate (one-quarter) of coaches and support staff who had completed formal training in mental health. Establishing basic mental health training to enable coaches to identify when an athlete may be at risk or experiencing symptoms of a mental health problem can assist in facilitating not only early intervention and treatment, but also increasing the likelihood of a faster recovery. This is of obvious importance to the athletes and coaches, as well as all other stakeholders; it is becoming increasingly more clear that athlete performance can be negatively impacted on by symptoms of mental disorders (Hill et al., 2015; Roberts et al., 2016).

The Sebbens et al. study indicated that coaches and other support staff may have had relatively high levels of depression literacy prior to the intervention. This could have been due to a self-selection bias, with those with higher literacy being more interested in participating. Nevertheless, mean confidence in assisting a person with a mental health problem at pre-intervention was only “moderate.” Thus, simply having an understanding of what depression is may not translate into feeling confident in identifying depression, feeling comfortable talking to someone with depression, or helping a person with depression find appropriate care. The program described in the current paper targeted those specific issues in a sport-specific context, which is likely to be highly useful to those completing the intervention in practical future situations.

Whilst it is notable that confidence in assisting a person with a mental health problem was improved after a brief workshop—does higher confidence in helping lead to increased helping behavior? We know that attitudes do not necessarily translate into behavior (Han et al., 2006). One previous study indicated that confidence in the ability of sport psychologists to assist athletes with both emotional problems and performance was highly predictive of *intentions* to use sport psychology consultations (Zakrajsek and Zizzi, 2007). However, studies of behavior are lacking, often because they are by nature required to be long-term studies with long periods of follow-up to determine if behavior change has occurred.

An avenue that remains unexplored was whether coaches have negative (stigmatizing) attitudes toward mental health problems. One of the key findings in a previous study (Gulliver et al., 2012) was the concern athletes expressed about what their coaches might think of them if they sought help for a mental health problem. There remains very limited research available on coaches' attitudes toward mental disorders (McArdle et al., 2016). It is important to reduce stigma of help-seeking for mental health problems in the general community, and specifically for elite athletes, a group who may feel scrutinized by their coach, peers, as well as the public.

Finally, it is also of note that the intervention instructors were both registered psychologists working in an elite sport organization. Actively facilitating the relationships between coaches, the athletes, and the mental health staff available to them is of vital importance. Specifically, the integration of psychologists into the athlete's support network may be crucial for expediting the pathways to care (Gulliver et al., 2012). It may be of benefit to have the program delivered by the psychologists available to the coaches and athletes to facilitate open dialog and establish highly functional working relationships amongst both staff and athletes.

In summary, the main findings of this study serve to explore and extend research into facilitating access to mental health care for athletes. Increasing discourse around athletes and mental health is crucial to eliminating stigma and viewing mental health as just another part of the athlete's overall health. Given the low rates of formal training noted in this study, translating this research into routine practice within sports is a high priority.

## Author contributions

The author confirms being the sole contributor of this work and approved it for publication.

### Conflict of interest statement

The author declares that the research was conducted in the absence of any commercial or financial relationships that could be construed as a potential conflict of interest.
